# Salivary beta-endorphin in nonsuicidal self-injury: an ambulatory assessment study

**DOI:** 10.1038/s41386-020-00914-2

**Published:** 2021-01-04

**Authors:** Lisa M. Störkel, Alexander Karabatsiakis, Johanna Hepp, Iris-Tatjana Kolassa, Christian Schmahl, Inga Niedtfeld

**Affiliations:** 1 Department of Psychosomatic Medicine, Central Institute of Mental Health, Medical Faculty Mannheim, Heidelberg University, Mannheim, Germany; 2 Department of Psychology, University of Innsbruck, Innsbruck, Austria; 3 Department of Clinical and Biological Psychology, Ulm University, Ulm, Germany

**Keywords:** Peptides, Diagnostic markers

## Abstract

Nonsuicidal self-injury (NSSI) is a prevalent and impairing behavior, affecting individuals with and without additional psychopathology. To shed further light on biological processes that precede and result from NSSI acts, we built on previous cross-sectional evidence suggesting that the endogenous opioid system, and especially β-endorphin, is involved in the psychopathology of NSSI. This is the first study assessing salivary β-endorphin in daily life in the context of NSSI acts. Fifty-one female adults with repetitive NSSI participated over a period of 15 days in an ambulatory assessment study. Salivary β-endorphin was assessed before and after engagement in NSSI, during high urge for NSSI, and on a non-NSSI day. Furthermore, NSSI specific variables such as pain ratings, as well as method, severity, and function of NSSI were assessed. We found that β-endorphin levels immediately before an NSSI act were significantly lower than directly after NSSI. However, there was no difference between β-endorphin during high urge for NSSI and post NSSI measures. We found a positive association between severity of the self-inflicted injury and β-endorphin levels, but no significant association between β-endorphin levels and subjectively experienced pain. The results of the present study indicate that it is possible to assess salivary β-endorphin in daily life in the context of NSSI. Furthermore, our results provide a first indication that NSSI acts could be associated with a momentary increase of β-endorphin, and this might reinforce NSSI engagement. More research is needed to replicate and extend our findings on peripheral β-endorphin in daily life.

## Introduction

Nonsuicidal self-injury (NSSI) is defined as the intentional and deliberate damage of one’s own body tissue without suicidal intent [1]. It is considered as a transdiagnostic symptom, but is particularly prevalent in affective disorders and borderline personality disorder (BPD) [2, 3]. Due to its high prevalence and marked negative outcomes, including increased risk of suicide or accidental death [4] and high associated health care costs [5], NSSI has been included as a new research diagnosis in the *Diagnostic and Statistical Manual of Mental Disorders* [1]. The pathogenesis of NSSI was repeatedly linked to prolonged experiences of psychosocial stress [6, 7], body objectification [8], or rejection or victimization by peers [9], potentially moderated by genetic predispositions [7].

In studies using self-report measures, those with NSSI indicated a reduction in negative feelings and aversive tension as their primary motive [10]. Therefore, theoretical models emphasize the role of negative reinforcement (e.g., escape from unwanted emotions) in the psychopathology of NSSI [11, 12]. Empirically, studies using ambulatory assessment (AA) demonstrated a reduction in negative affect and aversive tension following NSSI [2, 13]. Studies on (neuro-) biological underpinnings used NSSI proxies in the laboratory and found that individuals with NSSI, as compared to healthy controls (HCs), showed decreased subjective arousal [14] and a decreased heart rate in response to painful stimulation [15, 16]. Likewise, decreased amygdala activation through pain was observed in samples of BPD individuals with NSSI [as reviewed by [17]]. Finally, involvement of the endogenous opioid system (EOS) has repeatedly been discussed with regard to the development and maintenance of NSSI [18], mainly due to its role in the perception and regulation of social, emotional, and physical pain [18]. Peripherally released (conjugated) β-endorphin can pass the blood–brain barrier and influences the concentration of β-endorphin in the cerebrospinal fluid, whereas influence of peripherally released β-endorphin on concentrations in the central nervous system is limited [19, 20]. Furthermore, hormones in the central nervous system are able to initiate β-endorphin release in the periphery [21]. Finally, locally released β-endorphin (e.g., skin) modulates the perception of pain in the concerned area in addition to central mechanisms [22]. Taken together, it seems that peripheral as well as central systems are involved in the perception and regulation of pain [18].

In previous studies linking the EOS and NSSI, β-endorphin was the most investigated opioid for several reasons. First, tissue damage leads to secretion of peripheral β-endorphin in animals and humans [22, 23]. Second, lower peripheral levels of β-endorphin were found in humans with a history of NSSI during resting conditions [24], and in rhesus monkeys with a history of self-directed biting [25]. Third, there is also evidence for altered central β-endorphin and corresponding changes in µ-opioid receptor activity. One study assessed cerebrospinal fluid in individuals with personality disorders and found that those with a history of NSSI showed lower β-endorphin levels than those without [26]. In line with this, a study using positron emission tomography demonstrated that individuals with BPD and a history of NSSI had significantly more µ-opioid receptor availability than HCs. The authors interpreted this as indirect evidence for chronically low levels of β-endorphin in the concerned brain regions [27]. Fourth, low levels of β-endorphin were theoretically linked to dysphoria, inner emptiness and “the need to feel pain,” which are well known symptoms reported by self-injuring individuals [26].

Taken together, β-endorphin appears to be involved in the regulation of different forms of pain, and reduced β-endorphin levels were found in individuals with NSSI. Therefore, homeostasis model proposed by Stanley et al. [28, 29] proposes that NSSI acts may be a strategy to initiate the release of β-endorphin [18, 26, 30, 31]. However, previous studies on β-endorphin in NSSI were conducted in a laboratory context where individuals did not actually engage in NSSI. Thus, although previous work demonstrated that individuals with NSSI history differ from those without with regard to baseline levels of β-endorphin, further evidence for the assumption that NSSI is used to initiate the immediate release of β-endorphin is warranted, and can be tested by microlongitudinal assessment before and after NSSI acts.

## The present study

We used AA [32] to investigate the effect of NSSI on peripheral β-endorphin in daily life, using a smartphone-based application. Thereby, we focus on the question if NSSI could be used to initiate a release of β-endorphin by directly assessing the effect of real-life NSSI on the EOS, using a within subjects design. We chose to assess β-endorphin in saliva because participants are able to provide and store samples without interfering with daily activities.

In line with the theoretical assumption that individuals engage in NSSI to initiate a release of β-endorphin [18, 33], we hypothesized that (H1) peripheral β-endorphin levels are elevated immediately after engagement in NSSI, as compared to a saliva sample taken directly before NSSI (H1a), and as compared to a control condition during high NSSI urge but without engagement in NSSI (H1b). Given the reported association between β-endorphin and experience of physical pain [18, 34], we further hypothesized that (H2) higher levels of β-endorphin are associated with lower levels of experienced pain during NSSI. Based on findings that tissue damage leads to release of β-endorphin [22], we hypothesized that (H3) the severity of the injury is positively associated with β-endorphin levels.

## Materials and methods

### Participants

Participants were 51 women (aged 18–45, *M* = 23.92, SD = 6.72), recruited via flyers at local in- and outpatient clinics, by contacting patients on the waitlist of the Central Institute for Mental Health (CIMH) Mannheim, and via Facebook groups on NSSI-related topics. We recruited only women to reduce heterogeneity with regard to biological parameters. All participants met criteria for NSSI disorder according to the *Diagnostic and Statistical Manual of Mental Disorders* [1]. In addition, inclusion criteria were repeated engagement in tissue damaging NSSI for the last 3 months, with at least one NSSI incident per week. Exclusion criteria were current substance dependency, developmental disorders, schizophrenia, current pregnancy, medication influencing the EOS (e.g., naltrexone or other opioid analgesics), as well as exclusion criteria directly related to the assessment of salivary β-endorphin (e.g., frequent gum bleeding, see Supplementary Materials for details).

All participants provided written informed consent before participation and after they received a full description of the study protocol, which was approved by the ethics committee of the Medical Faculty Mannheim, Heidelberg University (2014-601N-MA). After participation, participants received 100€ for compensation, and an additional bonus of 50€ if they answered more than 80% of AA prompts.

### Procedure

Participants were invited to an on-site orientation session or an online orientation session (via the secured platform Patientus, jameda GmBH, Munich, Germany), which comprised clinical interviews (see “Measures”), self-report questionnaires^1^, an introduction to handling the saliva samples, and an introduction to the smartphone app (movisensXS, Version 0.7.4682, movisens GmbH, Karlsruhe, Germany) on the study smartphone. All participants were diagnosed by trained master’s level psychologists.

**Fig. 1:**
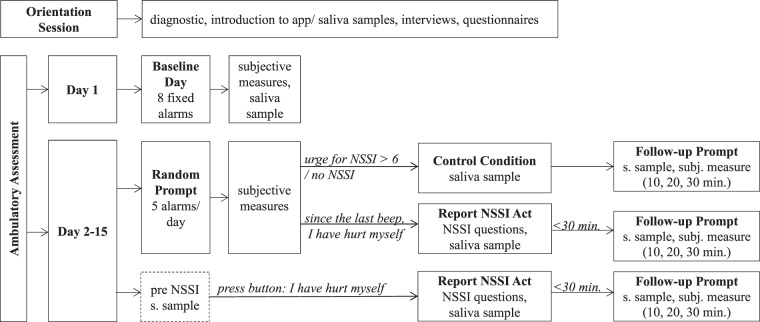
Study design: baseline day and random prompts assessed affect, interpersonal events, dissociation, tension, urge for NSSI, NSSI (yes/no), and control questions for β-endorphin. NSSI reports include NSSI specific questions about pain, method, motive, and severity. Control conditions followed a random prompt assessment and included a saliva sample. Follow-up prompts tracked the trajectory of affect, dissociation, tension, and pain, also including saliva samples. For a detailed description of assessments, see the “Measures” section and Supplementary Material.

The 15-day study period started with a baseline day in order to measure peripheral β-endorphin trajectory across a day without NSSI. On the baseline day, participants answered eight prompts (every 2 h) and provided a saliva sample at each timepoint. If participants engaged in NSSI (*n* = 8), the baseline day was repeated if possible, or saliva samples following the NSSI act were removed from the analyses. The following 14 days included five semirandomized prompts per day (self-reports without saliva sample; interval between prompts minimum 2 h) within participants’ normal waking hours. In addition, participants were asked to self-initiate a prompt as soon as possible following every NSSI act. Afterward, participants were asked to provide a saliva sample and answered NSSI-related questions (see “Measures”). After reporting an NSSI act, participants answered three follow-up prompts (after 10, 20, and 30 min), also including a saliva sample for each timepoint (see Fig. 1). In addition, we asked participants to provide a saliva sample shortly before they engaged in NSSI, if possible. However, saliva samples before NSSI were not implemented in our smartphone app design to keep participant burden low. Finally, if participants reported a high urge for NSSI (>6 on a 0–10 scale) during a random prompt, but did not yet engage in NSSI, they were asked to provide a saliva sample for a control condition. In the next 30 min, participants answered three follow-up prompts, parallelized with the NSSI follow-up prompts. To keep participant burden as low as possible, this control condition occurred only as frequently as NSSI acts occurred. Besides NSSI acts, urges, pain, and salivary β-endorphin levels, we also assessed momentary affect, dissociation, and interpersonal stressors^2^.

### Measures

#### Sociodemographic data

We assessed age, body mass index, years of school education, current employment status, and current medication (Table 1). The majority of our sample (*n* = 30; 58.82%) reported intake of permanent psychiatric medication, with antidepressants (*n* = 30) and atypical antipsychotics (*n* = 14) as the most common ones^3^. We also asked participants about their daily physical activity, sports, and possible gum bleeding, which are known confounders in the analysis of saliva samples [35].

**Table 1:** Demographic and clinical characteristics.

Characteristic	*n*	%	Range	Mean	SD
Demographic variables					
Body mass index	51		17.2–34.4	24.2	4.9
Years of education	51		8–15	11.87	1.44
Employment status	51				
Employed	17	33.33			
Student or pupil	16	31.37			
Unemployed	14	27.45			
Disability pension	4	7.84			
History of nonsuicidal self-injury^a^					
Age of onset	50		6–28	14.33	3.86
Estimated lifetime NSSI	49		25–2590	763.82	664.03
Past year	50		25–624	126.48	103.72
Past month	50		3–32	10.36	6.44
Engagement of years in NSSI	50		0–33	9.65	6.48
Comorbid diagnoses^b^					
Mood disorders					
Major depression	33				
Dysthymia	4				
Anxiety disorders					
Social phobia	11				
Specific phobia	6				
Generalized anxiety disorder	2				
Panic disorder	6				
Agoraphobia without panic	2				
Posttraumatic stress disorder	25				
Obsessive comp. disorder	6				
Substance use disorders					
Substance abuse	2				
Somatic disorders					
Somatic pain disorder	1				
Eating disorders					
Anorexia	6				
Bulimia	5				
Attention deficit disorder	1				
Borderline personality disorder	32				
Any mental disorder	51		0–5	2.24	1.45

^a^Questionnaire data of the self-injurious thoughts and behavior interview: German.

^b^Diagnosis according to SKID-I and IPDE.

#### Clinical interviews

To assess current and past psychopathology, the structured clinical interview for the *Diagnostic and Statistical Manual of Mental Disorders for Axis 1* [36] was administered. We also administered the BPD section of the International Personality Disorder Examination [37]. On average, participants had 2.24 (SD = 1.45) comorbid diagnoses (Table 1). We used the Self-Injurious Thoughts and Behavior Interview (SITBI-G, [38]) to assess NSSI diagnosis, frequency, and methods.

### AA measures

#### Nonsuicidal self-injury

Following each NSSI act, participants reported how much time passed by since they self-harmed (in minutes), the method used (e.g., cutting), motives for NSSI (e.g., “reduce tension”), and the effectiveness of NSSI (“yes,” “no,” “I don’t know”). They were also asked to self-rate the severity of the wound as “mild” (superficial cuts, bruise, scratching), “moderate” (not only skin, but also underlying tissue is damaged, strongly bleeding cuts, second/third degree burns), or “severe” (cuts to fat tissue, damaged sinews, bone fractures, inner bleeding). They reported on current pain intensity, pleasantness/unpleasantness of current pain, and pain during NSSI (each on an 11-point Likert scale, ranging from “no pain” (0) to “worst imaginable pain” (10) or “pleasant” (0) to “unpleasant” (10)). A detailed overview of all AA items and answer options is presented in Supplementary Material.

#### Urges for NSSI

This was assessed via the single item “during the last 15 min the urge to hurt myself was” on a visual analog scale from “no urge at all” (0) to “I can hardly contain myself” (10).

#### Control questions

To minimize confounds with regard to β-endorphin, we asked participants at the end of each prompt, including a saliva sample, if they had used drugs/alcohol, had sex, or did sports within the 1.5 h before sampling. If one of these options was answered with “yes,” the respective saliva sample was excluded from analyses (*n* = 52).

#### Saliva samples

Participants were instructed to put the synthetic swab of the saliva sample (Salivettes^®^, code blue, Sarstedt, Germany) into their mouth without using their hands, and chew the swap slightly for 30 s. Next, they were asked to translocate the swab directly back into the collection tube and freeze the sample immediately in their own freezer (at least −18 °C/−0.4 °F). After completion of the study, frozen tubes were collected from participants’ homes and transported to the CIMH Mannheim using dry ice. Saliva samples were stored at the BioPsy Biobank of the Department of Genetic Epidemiology in Psychiatry at the CIMH Mannheim [39] at −80 °C (−112 °F) for up to 22 months^4^.

### Data analysis

#### Biological data

We analyzed salivary β-endorphin using 15 ELISA kits (Cat. No. S-1134; Peninsula Laboratories International, San Carlos, CA, USA) with the same LOT number. All samples were thawed for 2.5 h at 4 °C (39.2 °F) in a refrigerator prior to centrifugation at 3000 × *g* for 10 min. Saliva aliquots were analyzed using ELISA following protocol III of the manufacturer’s manual (peptide enzyme immunoassay protocols; Peninsula Laboratories International, San Carlos, CA, USA; see Supplementary Material). ELISA plates were measured using a TECAN M400 ELISA plate reader, connected to a PC running the operating software MAGELAN (Tecan International, Germany). As the expected range of β-endorphin levels in saliva were not clearly defined in the literature, we decided to extend the range of the standard curve by adding two additional concentrations at the higher end (except for the first plate analyzed). The new standard curve now covered a concentration between 0.08 and 100 ng/ml. For the calculation of the standard curve and the slope function, we used the calculation sheet provided by the manufacturer of the kit.

#### Statistical analysis

For the analysis of β-endorphin (ng/ml), we used log-transformed values to reduce skewness of the data. To account for the nested data structure in AA, we employed multilevel models (MLMs). We modeled random intercepts per participant and random slopes for central predictors (but not covariates) and performed all analyses in R, using the lmer and glmer functions from the package lme4 [40, 41].

## Results

Participants completed a total of 4619 prompts, which is an average of 90.57 prompts (SD = 19.65) per participant, resulting in a high compliance rate of 92.04%. One participant lost the study smartphone (providing 60 data points), and two participants quit participation prematurely because they accepted an elective residential treatment unrelated to the present study (32 and 21 data points, respectively). All available data points were used for subsequent analyses.

All participants cumulatively provided 1162 saliva samples (*M* = 23.24, SD = 11.14) (see Table 2 for descriptive data on β-endorphin). One participant did not return the saliva samples. We removed six saliva samples because they could not be assigned to app data due to wrong code input by participants. Three participants accidently completed two baseline days, so we removed the second baseline day from analysis (*n* = 18). Eight participants reported NSSI engagement on baseline day, so we removed saliva samples following the NSSI event (*n* = 24). Furthermore, 49 saliva samples were excluded because the β-endorphin concentrations were above the maximum of the standard curve of the ELISA (*n* = 5), or because participants reported sports activities (*n* = 43) or sexual activity (*n* = 1) 1.5 h before providing the saliva sample.

**Table 2:** Characteristics of NSSI acts.

Variable	*n*	%	Mean	SD	Range
Method					
Cutting	107				
Wound manipulation	28				
Scratching	19				
Burning/ice burning	9				
Head banging/punching self	4				
Other	2				
More than one method	14	8.28			
Motive					
To reduce aversive tension or overwhelming emotions	99	45.81			
Self-hatred/self-contempt	59				
To feel something (other than nothing)	31				
Help/attention of others	8				
Other reason	20				
I don’t know why I self-harmed	9				
More than one motive	71				
Severity of NSSI^a^					
Mild	47	31.76			
Moderate	88	59.46			
Severe	13	8.78			
Mean painfulness for severity of the wound^b^					
Mild			1.8	2.08	
Moderate			2.55	1.76	
Severe			4.36	2.5	
β-endorphin (ng/ml)^c^					
Pre NSSI	18		11.65	10.82	
Post NSSI	476		13.94	11.19	
Control condition	236		12.6	12.29	
Baseline day	333		14.33	15.47	
Variability within person			9.45	7.74	1.09–45.86
Variability between person			14.07	14.26	0.11–161.55

^a^Severity categories: mild: superficial cuts, bruise, and scratching; moderate: not only skin, but also the underlying tissue is damaged, strong bleeding cuts, and 2/3 grade burning; and severe: cuttings until fat tissue, damaged sinews, bone fractures, and inner bleeding.

^b^Painfulness was rated on an 11-point Likert scale from 0 (no pain) to 10 (worst imaginable pain).

^c^Raw mean values of β-endorphin in ng/ml.

Participants reported 155 NSSI acts, which equates to an average of 3.04 NSSI acts per person (range 0–15), and completed a total of 391 NSSI follow-up prompts. Participants reported NSSI acts after 1–40 min (*M* = 6.83, SD = 5.75). For our analyses, we excluded NSSI acts that were reported later than 30 min post NSSI (*n* = 11) due to the enzymatic degradation of β-endorphin in saliva under room temperature [42]. For the control condition, participants answered 109 prompts with 261 follow-up prompts. Furthermore, participants were able to provide saliva samples before NSSI acts in 18 cases, on average 8.89 min (SD = 3.49) before they engaged in NSSI. After the above-mentioned exclusions, 1054 saliva samples were included in our final analysis.

### Descriptive statistics for NSSI data

Cutting was the most frequent NSSI method (*n* = 107), and the most endorsed reason for NSSI was “to reduce aversive tension/overwhelming emotions” (*n* = 99). Participants rated 148 NSSI acts with regard to severity (see Table 2). In most cases, they rated NSSI severity as “moderate” (59.46%). Over all three categories of wound severity, participants indicated rather mild pain (*M* = 2.26; SD = 2.08). More specifically, in 71.32% of NSSI acts, participants reported that they felt no or very mild pain.

### Baseline day trajectory

To assess the trajectory of β-endorphin across the day, we predicted β-endorphin levels in two MLMs with the participants’ wake time in (a) hours and (b) the time of day as predictors, modeling random slopes for these predictors. Results showed that β-endorphin levels did not vary systematically across participants’ wake times (Est. = −0.02, SE = 0.02, *p* = 0.456, *β* = −0.03, CI [−0.12, 0.05]) nor across time of day (Est. = −0.01, SE = 0.01, *p* = 0.473, *β* = −0.03, CI [−0.12, 0.05]). Therefore, these variables were not included as covariates in the following models.

### Momentary β-endorphin

**Fig. 2:**
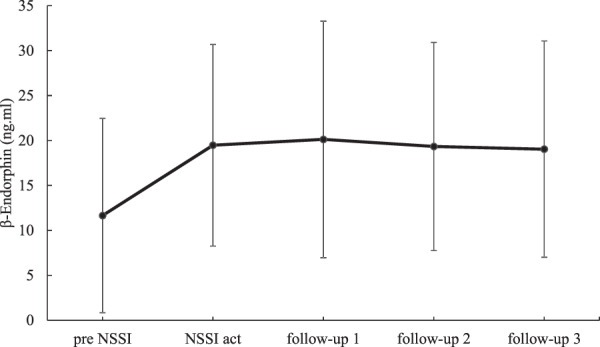
Trajectory of salivary β-endorphin (ng/ml) from pre NSSI to post NSSI for the subsample, only including participants who provided a pre NSSI saliva sample (*n* = 18). Pre NSSI samples were provided on average 8.89 min before the NSSI act. Time intervals between the report of the NSSI act and the follow-up prompts are 10 min each. Standard deviations are represented in the figure by the error bars attached to the line.

To test hypothesis H1a that β-endorphin in saliva is elevated directly after NSSI acts, as compared to samples collected directly before NSSI, we conducted an MLM, including only participants who provided a saliva sample prior to NSSI (pre NSSI samples: *n* = 18, post NSSI samples: *n* = 37, follow-up samples: *n* = 104). We predicted β-endorphin levels with a pre–post NSSI dummy variable (pre = 0, post = 1). Results indicated that β-endorphin levels were significantly higher in the post versus the pre NSSI conditions (Est. = 0.62, SE = 0.2, *p* = 0.032, *β* = 0.21, CI [0.07, 0.34]) (see Fig. 2). Further specifying the effect size, Cohen’s *d* [43] with regard to a paired *t*-test was large (*t* = 3.67, *p* = 0.001, *d* = 0.82), and a Bayes factor [44] of 21.49 also indicated strong evidence for a difference between pre and post NSSI samples.

**Fig. 3:**
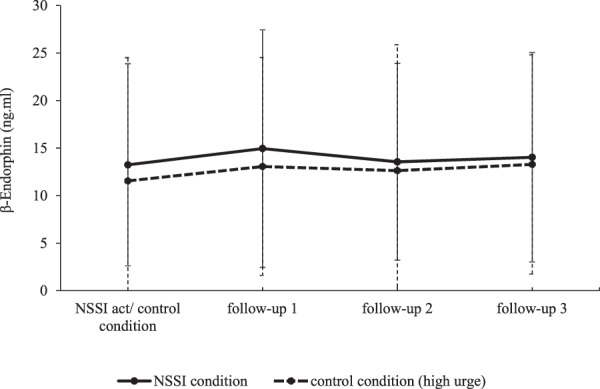
Trajectories of salivary β-endorphin (ng/ml) for the NSSI and the control condition. Time intervals between the first prompt and the follow-up prompts are 10 min each. Standard deviations are represented in the figure by the error bars attached to each line.

Next, we computed an MLM to compare saliva samples collected after NSSI to the control condition (H1b). Here, we predicted β-endorphin levels with a dummy variable coding post NSSI samples as 0 and control condition samples as 1 (see Fig. 3). Results showed no significant differences between these conditions (Est. = −0.03, SE = 0.11, *p* = 0.766, *β* = −0.01, CI [−0.1, 0.07]), indicating that during high urge for NSSI, β-endorphin was not significantly lower than directly after NSSI. In an additional exploratory analysis, we also found no difference between post NSSI samples and β-endorphin levels on baseline day (i.e., non-NSSI day) (Est. = −0.01, SE = 0.08, *p* = 0.938, *β* = 0.0024, CI [−0.07, 0.06]).

To test hypothesis 2, we predicted subjective pain following NSSI with β-endorphin levels, while modeling a random slope for the β-endorphin predictor. Contrary to our hypothesis, higher salivary β-endorphin did not entail lower levels of experienced pain in the 30 min following NSSI (Est. = 0.4, SE = 0.31, *p* = 0.199, *β* = 0.1, CI [−0.05, 0.24]). However, even though participants rated more severe wounds as significantly more painful (Est. = 1.14, SE = 0.48, *p* = 0.03, *β* = 0.33, CI [0.06, 0.6]), they reported rather mild pain overall, leading to low variance in pain ratings.

Finally, to test the association between injury severity and β-endorphin levels (hypothesis 3), we predicted β-endorphin levels with severity (−1 = mild, 0 = moderate, 1 = severe), again modeling a random slope for severity. We found a positive association between injury severity and levels of β-endorphin (Est. = 0.39, SE = 0.15, *p* = 0.009, *β* = 0.2, CI [0.05, 0.35]), indicating that more severe injuries were associated with greater β-endorphin release.

## Discussion

The present study evaluated potential effects of NSSI on the EOS in daily life. As hypothesized, we found that NSSI had a significant and large effect on β-endorphin levels in individuals with chronic NSSI. More specifically, we found that immediately before NSSI, β-endorphin levels were significantly lower as compared to post NSSI samples. This finding supports theoretical assumptions of the homeostasis model of NSSI [26, 31], specifically that individuals use NSSI to return to their intraindividual norm-physiological β-endorphin range [18, 26, 30]. Furthermore, our study extends seminal previous work [26], by assessing momentary activity of EOS in individuals with NSSI.

Contrary to our hypothesis, we did not find a significant difference in β-endorphin levels between post NSSI samples and a control condition with a high urge for NSSI. This is not in line with the assumption that low levels of β-endorphin are accompanied by high urges for NSSI [26]. However, our finding may be attributable to the relatively small number of saliva samples that were collected during very high levels of urge (*n* = 32). Furthermore, control conditions only occurred when urge was between 7 and 10 on an 11-point Likert scale, resulting in restricted variance in the urge variable (*M* = 7.59, SD = 0.82). Due to limited sample size and restricted variance, we were not able to test the relationship between β-endorphin and NSSI urge, based on our current sample. Future research could systematically assess the relationship between urge and β-endorphin to asses if low β-endorphin levels are uniquely associated with NSSI urges. However, we also did not detect significant differences when comparing post NSSI samples with a non-NSSI baseline day in an exploratory analysis. Taken together, we found no indication for higher-than-usual levels of β-endorphin directly after NSSI. Therefore, we conclude that one reason for the engagement in NSSI could be the release of β-endorphin to restore homeostasis, which is in line with previous theoretical assumptions [18, 26, 30].

With regard to the relationship between tissue damage and changes in β-endorphin levels, we found a positive association between severity of the self-inflicted injury and levels of momentary β-endorphin, which is in line with previous research [22, 23]. To the best of our knowledge, no study previously assessed the correlation between β-endorphin in saliva and in other peripheral biofluids (e.g., blood, urine, cerebrospinal fluid). Therefore, our raw values cannot be quantitatively compared to studies assessing β-endorphin in other peripheral biofluids.

We did not observe a significant association between salivary β-endorphin concentration and subjective pain ratings. Individuals in our sample frequently reported either analgesia or mild pain during NSSI. Even though more severe wounds were rated as significantly more painful and were associated with higher levels of β-endorphin, participants rated all three categories of severity with low-to-moderate painfulness. On the one hand, restricted variance in the pain variable may have caused these nonsignificant findings. On the other hand, the subjective experience of pain may be modulated by top-down cognitive processes [45, 46], in addition to β-endorphin response in the periphery. Thus, future studies should assess central mechanisms of pain regulation, and combine this with measures of β-endorphin. Nevertheless, our findings on the effect of injury severity indirectly support previous assumptions of analgesic effects of β-endorphin [11, 18, 34], and extend these findings to daily life. Notably, reduced pain sensitivity is related to repetitive engagement in NSSI [47], possibly due to the absence of negative consequences of the harmful behavior.

In line with findings from a study assessing salivary β-endorphin in the morning and evening [48], we did not find a circadian trajectory of β-endorphin in our sample. This simplifies the interpretation of our data at the momentary level.

### Limitations

This study was the first with a microlongitudinal AA design that allowed assessing the immediate effects of NSSI acts. It demonstrated that a noninvasive assessment of β-endorphin via saliva samples is possible in daily life and provides a methodological basis for future testing of the EOS theory in daily life. However, our study design has some limitations that should be improved in following research. First, although the current sample comprised 155 NSSI episodes with saliva samples post NSSI, which is comparable to previous studies in daily life [2], our main result is based on 18 saliva samples that were provided immediately before an NSSI act. Although β-endorphin increase from pre to post NSSI was a large effect, statistical power is limited by the small number of saliva samples. Since our study shows that participants were able to provide pre NSSI samples, future studies should systematically include pre NSSI saliva samples, as well as pre NSSI self-ratings (e.g., urge, affect), to enhance the understanding of the impact of NSSI on the EOS.

Second, participants self-administered the saliva samples. While we assessed several potential confounders and removed respective prompts from the analyses, saliva samples may still have been influenced by a range of other internal or external factors (e.g., food, freezer temperature, tobacco, stress). This could have reduced the reliability of the β-endorphin assessment and introduced large standard errors in the models. Evidently, this was a direct result of sampling in daily life and is a limitation that has to be weighed against the strengths of sampling real-life data. Finally, we only focused on intrapersonal changes of β-endorphin. Future research is needed to replicate and extend our findings, especially by including a control group without NSSI history to test between-person differences of β-endorphin in daily life.

## Conclusions

The present study was the first to demonstrate that a noninvasive assessment of β-endorphin levels in daily life is possible and feasible via saliva samples. Our findings indicate that momentary changes in β-endorphin are potentially involved in the psychopathology of NSSI. First, levels of salivary β-endorphin were reduced immediately before NSSI, as compared to post NSSI samples, suggesting a return to normal β-endorphin levels by means of NSSI. Second, more severe tissue damage was associated with higher levels of β-endorphin. Further research is needed to replicate and extend our findings, especially with regard to reduced β-endorphin shortly before NSSI.

## Funding and disclosure

This research was supported by the Central Institute of Mental Health (CIMH) Young Investigator Award of the CIMH Mannheim awarded to IN. Authors LMS, AK, JH, I-TK, and IN declare no potential conflict of interest. CS received advisory panel payments and research grants from Boehringer Ingelheim International GmbH. Open access funding provided by Open Access funding enabled and organized by Projekt DEAL.

## Supplementary information

Suppelemental Material

## Footnotes

1 Not part of this article, but a list of the questionnaires is included in the Supplementary Material.

2 Results of the latter aspects are not subject of this paper.

3 Including medication as a control variable did not change the results of our models.

4 Including storage time (in months) as a control variable did not change the results of our models.
