# Supplementary material for: Salivary beta-endorphin in nonsuicidal self-injury: an ambulatory assessment study
Source: Neuropsychopharmacology. 2021 Jan 4;46(7):1357–63. doi: 10.1038/s41386-020-00914-2 (PMC8134499; doi:10.1038/s41386-020-00914-2)
Supplement: Supplementary file 1 — Suppelemental Material [file 41386_2020_914_MOESM1_ESM.docx]

**Supplemental Material: Salivary beta-endorphin in non-suicidal self-injury: an ambulatory assessment study**

1. **Sample**

- 51 women (aged 18-45, *M*=23.92, *SD*=6.72)
- *Inclusion criteria:* aged between 18-45, NSSI according to DSM-5 (and ≥1 NSSI acts per week for the last three months), female.
- *Exclusion criteria:* Life-time diagnosis of schizophrenic disorders, mental retardation/developmental disorders, substance dependency within the last 6 months, current injuries not related to NSSI (e.g. slipped disc, operation etc.), BMI < 17.5 or > 34, pregnancy, current use of cannabis or other stimulating drugs, and medication with opiates, naltrexone, cortisone, opioid analgesics. Additional exclusion criteria related to the collection of saliva samples were frequent gum bleeding (e.g. gum bleeding while brush one’s teeth), tooth or root canal treatment in the last 2 weeks, no access to a freezer (at least -18°C/-0.4°F).

1. **Orientation session:**

Clinical Interviews:

*Structured Clinical Interview for DSM-IV (SCID-I)* [1]. Borderline Personality Disorder section of the *International Personality Disorder Examination (IPDE)* [2].

*Self-injurious thoughts and behavior interview: German* [SITBIG, 3]. The SITBIG is a semi-structured interview assessing thoughts, affect, motives and interpersonal problems concerning suicidal tendencies and NSSI. Furthermore, it captures frequency, methods, and severity of NSSI.

Self-report questionnaires:

*Screening Questionnaire for DSM-IV Axis II Diagnosis (SCID-II).* The SCID-II screening questionnaire [4] is a 117-item forced choice (yes/ no) self-report questionnaire, assessing indications of axis II personality disorders according to DSM-IV. The questionnaire addresses behavior, experiences, and beliefs over the last ten years, showing long lasting patterns of personality.

*Questionnaire for Assessment of the Severity of NSSI (QASN; Landau).* The QASN is a not yet validated German self-report questionnaire, assessing frequency, methods, body parts, severity, motives, and impulsivity of NSSI. Furthermore, it assesses urges for NSSI and captures if participants are willing to reduce NSSI.

*Assessment of pain during NSSI:* Participants were asked on a scale from *no pain (0)* to *highest imaginable pain (10)* if they: 1) Feel pain during NSSI in general, 2) Felt pain the last time they engaged in NSSI, 3) Felt pain the first time they engaged in NSSI, 4) Felt pain when they began damaging body tissue, 5) Felt pain in the minutes after engaging in NSSI.

Furthermore, we asked participants: “How long does it take, on average, until your feeling of pain returns to normal after engaging in NSSI?” The answer options were: a) *“my feeling of pain is not different during NSSI”, b) “ten minutes or less”, c)“ten to thirty minutes”, d)“thirty minutes to an hour”, e) “one hour to one day”, f) “more than one day”.*

We further asked participants: “Has the intensity of physical pain during NSSI changed since the first time you engaged in NSSI?” The answer options were: a)*“Now, I feel muss less pain than during my first NSSI”, b) “Now, I feel a little less pain than during my first NSSI”, c)“I feel the same amount of pain during NSSI than during my first NSSI”, d) “Now, I feel a little more pain than during my first NSSI”, e) “Now, I feel much more pain than during my first NSSI”*.

*Suicidal thoughts and behaviors:* We also added questions to assess suicidal thoughts and behaviors, asking participants if they: 1) had ever thought about suicide, 2) had ever made a suicide attempt, if yes 3) how many suicide attempts they had made and 4) when they had made the most recent suicide attempt.

*HEXACO Personality Inventory.* The German version of the HEXACO [5] is a 60-item self-report questionnaire, assessing the personality dimensions Honesty-Humility, Emotionality, Extraversion, Agreeableness, Conscientiousness, and Openness to Experience with a five-point Likert scale from *strongly disagree (1)* to *strongly agree (5)*.

*Borderline Symptom List (BSL-23).* The German adaption of the BSL-23 [6] is a 23-item self-rating scale, based on the criteria of the DSM-IV (revised version), to assess core symptoms of the Borderline Personality Disorder. Individuals answer on a five-point scale from *not at all (0)* to *absolutely true (4)*.

*Dissociation Tension Scale (DSS).* The German version of the DSS [7] is a 21-item self-report questionnaire, assessing dissociative symptoms over the last seven days, using an 11-point Likert scale from *no* (0%) time of the day to *always* (100%) during the day. Another single item assesses frequency of aversive tension during the last seven days on the same scale.

*Childhood Trauma Questionnaire (CTQ).* The German version of the CTQ [8] is a retrospective self-report questionnaire, screening for childhood maltreatment (sexual, physical, and emotional abuse as well as physical and emotional neglect), using a five-point Likert scale from *not at all (1)* to *very frequently (5)*.

*Social Network Index (SNI).* The German version of the SNI [9] is a 12-item self-report questionnaire, assessing quality and quantity in twelve different types of social interaction in daily life. It addresses the different social roles of the participants, as well as social loneliness and diversity and size of social networks.

*Potentially confounding Variables:* Menstruation cycle (days), smoking behavior (yes/ no and cigarettes per day), daily physical activity (sports) (in minutes).

1. **Ambulatory Assessment sampling scheme**

Participants completed 15 days of Ambulatory Assessment (AA). First, they completed a baseline day with 8 prompts every two hours (self-report and saliva sample), and then participants completed 14 study days. During the 14 study days, sampling was as follows:

- 5 semi-random (> 2h apart) prompts (self-report data)
- self-initiated prompts after NSSI act, entailing self-reports and saliva sample, with three follow-up prompts (10, 20 and 30 minutes after report of NSSI act), assessing self-reports and saliva samples
- control condition with high urge for NSSI (>6 on visual analog scale 0= no urge at all to 10=I can hardly contain myself), entailing self-reports and saliva sample, with three follow-up prompts (10, 20 and 30 minutes after report of high NSSI urge), assessing self-reports and saliva samples

1. **Ambulatory Assessment Items**

Random prompts (five pseudo-randomized prompts per day, >2h apart):

*Momentary affect:* We assessed mood and current emotions to capture momentary affect. Current mood (“At the moment, I feel….”) was assessed by *Multidimensional Mood Questionnaire (MDMQ)* [10], using the items tired-awake, content-discontent, agitated-calm,  full of energy-without energy, unwell-well, relaxed-tense (bipolar scale: +++, ++, +, 0, -, --, ---). Thirteen items from the *Positive and negative Affect Scale* [PANAS-X, 11] were used to assess positive affect (“At the moment, I feel….”) via the items daring, attentive, delighted, bold, happy, and concentrating (Likert-scale, 1-5). Negative affect was assessed with the items disgusted with self, loathing, downhearted, afraid, hostile, nervous, and blameworthy (Likert-scale, 1-5). Additionally to the PANAS-X-items, we included two items *dead inside* and *empty inside* to capture feelings of emptiness, which are discussed with regard to NSSI [12,13] and were also described as a symptom of borderline personality disorder [14]. To reduce patient burden, two items of each scale of the PANAS-X [11] were selected for our study, based on factor analysis after evaluation in an online study (for more details on online study, see paragraph on interpersonal events).

*Dissociative symptoms:* We assessed dissociation via the Dissociation Tension Scale (DSS-4) [15]. Participants answered four items (“At the moment I have the impression that….”) on a 10 point Likert scale from 0 = “not present” to 9 = “very strong”. 1) “My body does not belong to me” (depersonalization), 2) “I have problems hearing, e.g. I hear sounds from nearby as if they come from far away” (somatoform dissociation), 3) “Other people or things around me are unreal” (derealization), “My body or parts of it are insensitive to pain” (analgesia).

*Interpersonal events:* Participants indicated significant interpersonal events (“Since the last prompt, another person…”) with positive and negative valence (checkboxes with multiples possible answers). For positive events, they could choose one of the following options: a) “supported/helped me”, b) “showed me affection”, c) “respected my needs or feelings”, d) “gave me their attention or time”, e) “was interested in me or took me seriously”, f) “none of the above”. If any event was endorsed, they additionally answered questions on the impact (“What the person did distressed me”, 0 = “not at all” to 5 = “very deeply”) and relation to NSSI (“What the person did was a reaction to my last NSSI”, “yes”, “no”, “I don´t know”). Similarly, participants were asked to indicate negative interpersonal events (checkboxes with multiples answers possible) with the following options: a) “criticized me”, b) rejected me/ excluded me”, b) “ignored my needs or feelings”, c) “behaved angry or aggressive towards me”, d) “let me down/ disappointed me”, e) “none of the above”. If any event was endorsed, they additionally answered questions on the impact (“What the person did distressed me”, 0 = “not at all” to 5 = “very deeply”) and relation to NSSI (“What the person did was a reaction to my last NSSI”, “yes”, “no”, “I don’t know”).

Items for positive and negative interpersonal events were chosen based on an online survey with 376 participants. Participants were aged between 18 – 65 (*M* = 30.2, *SD* = 10.2), the majority of the sample was female (*n* = 283), and many fulfilled the clinical cut-off for borderline features (*n* = 119) in the German version of the *Personality Assessment Inventory* [16,17]. During this pilot study, participants were asked to describe one positive and one negative interpersonal event they experienced with a significant other person during the last seven days. After that, they were asked to retrospectively rate their emotions for each of the two previously described interpersonal events with the 60 items of PANAS-X [11]. In a next step, participants were asked to rate each event (positive and negative) on nine different categories. We used the five most commonly endorsed categories for positive and negative events in the current study.

*Non-suicidal self-injury (NSSI):* “Since the last beep I answered, I have hurt myself.” (“yes” / “no”).

*Urge for NSSI:* “During the last 15 minutes the urge to hurt myself was” (visual analog scale: 0 = “no urge at all”, 10 = “I can hardly contain myself”).

Optional items for random prompts, whenever participant indicated an NSSI event:

*Time since NSSI:* “Since I have hurt myself, XX minutes passed by” (sliding wheel, list of minutes).

*Saliva sample:* Participants were instructed as follows. Screen one: “Please flush your mouth with water and make sure that you don’t have food left over in your mouth. Please wait until your salivation is normal again. Please try not to smoke, eat, or drink in the next 30 minutes. Now, put the swab from the collection tube in your mouth without using your hands. Once you have the swab in your mouth, please report the number on the collection tube. Press the “continue” button to start the timer (30 seconds). Screen two: “For the next 30 seconds, please chew the swab slightly to stimulate your salivation. Keep the swab in your mouth until you have the impression that the swab is saturated with saliva. This is very important for our study!” Screen three: “Spit the swab back into the collection tube, without using your hands, and put the collection tube back in the larger tube. Please close the collection tube with the cap. Now, put the collection tube in your freezer as fast as possible. Thank you for your participation!”

*NSSI method:* “I have hurt myself through…” (checkboxes/multiple answers possible) cutting, wound manipulation, scratching, burning/ ice burning, head banging/ punching self, other.

*NSSI motives*: “I have hurt myself because I…” (checkboxes/ multiple answers possible) wanted to reduce aversive tension or overwhelming emotions, wanted to express my self-hatred/ self-contempt, wanted to feel something (other than nothing), wanted help/ attention of others, had another reason, don’t know why I self-harmed.

*NSSI effectiveness*: “Did the NSSI act have the desired effect?” (forced choice: “yes”, “no”, “I don’t know”)

*NSSI severity*: “The severity of my wound is...” (forced choice): Mild/ superficial wound (superficial cuts, bruise, scratching), Moderate wound (not only skin, but also underlying tissue is damaged, strongly bleeding cuts, 2^nd^/3^rd^ degree burns), Severe wound (cuts to fat tissue, damaged sinews, bone fractures, inner bleeding).

*Intensity/ painfulness during NSSI*: “During self-injury, the intensity of pain was…“ (visual analog scale: 0 = “no pain”; 10 = “worst imaginable pain”).

*Pleasantness of pain during NSSI*: “During self-injury, the pain was...” (visual analog scale: 0 = “pleasant”; 10 = “unpleasant”)

*Actual pleasantness of pain*: “At the moment pain is…” (visual analog scale: 0 = “pleasant”; 10 = “unpleasant”)

*Actual intensity/ painfulness*: “At the moment, intensity of pain is…” (visual analog scale: 0 = “no pain”; 10 = “worst imaginable pain”)

*Control questions*: To assess possible confounders of -endorphin, participants indicated “In the last 1, 5 hours I have…” (checkboxes/ multiple answers possible) done sport, consumed drugs, consumed alcohol, had sex, nothing of the above.

NSSI report (self- initiated):

In case of an NSSI event, participants were asked to self-initiate the app as soon as possible. During the event-related prompts, the following information was assessed (for full list of items, see paragraph on random prompts above): Time since NSSI, saliva sample, NSSI method, NSSI motive, NSSI effectiveness, NSSI severity, intensity/ painfulness during NSSI, pleasantness of pain during NSSI, actual pleasantness of pain, actual intensity/ painfulness, momentary affect (MDBF, PANAS), dissociative symptoms (DSS-4), interpersonal events, control question.

Each NSSI event triggered three follow up prompts (10, 20 and 30 minutes later), each entailing a saliva sample, actual pain intensity/ actual pain valence, momentary affect (MDBF, PANAS), and dissociative symptoms (DSS-4). For (for full list of Items, see paragraph on random prompts above).

Control condition:

If participants reported an NSSI urge > 6 (0 = “no urge at all”, 10 = “I can hardly contain myself”) during a random prompt, but did not engage in NSSI, a control condition was triggered. To reduce patient burden, control conditions occurred only as frequently as NSSI acts. Control conditions comprised of a saliva sample and control questions (for full list of Items, see paragraph on random prompts above).

Each control condition triggered three follow up prompts (10, 20 and 30 minutes later), entailing a saliva sample, and assessment of momentary affect (MDBF, PANAS) and momentary dissociation (DSS-4). For full list of Items, see paragraph on random prompts above.

1. **Processing of Saliva Samples**

We used Protocol III (Std. Ab1hr.Bt) for the analysis of salivary beta-endorphin in NSSI, as provided by the manufacturer of the ELISA kits (Cat.No. S-1134; Peninsula Laboratories International, San Carlos, USA).

**1 – Into each well of the immunoplate add**

**50 µl standard or sample** (in diluent)

**25 µl antiserum** (in EIA buffer)

Add 50 µl diluent and 25 µl EIA buffer to blank wells.

**2 – Incubate at room temperature for 1 hour.** Shorter pre-incubations may result in lower sensitivity.

**3 – Rehydrate the Bt-tracer** (in EIA buffer) **and add 25 µl / well.**

**4 – Incubate at room temperature for 2 hours.**

**5 – Wash immunoplate 5 times with 300 µl/well of EIA buffer.** Be very careful not to cross-contaminate between wells in the first wash/ dispensing cycle. In each wash cycle empty plate contents with a rapid flicking motion of the wrist, then gently blot dry the top of the plate on paper towels. Dispense 300 µl of EIA buffer into each well and gently shake for at least a few seconds. Thorough washing is essential.

**6 – Add 100 µl/ well of streptavidin-HRP.** Trap or centrifuge the SA-HRP vial to collect all liquid contents on the bottom of the vial. Dilute 1/200 in EIA buffer (60 µl /12ml) and vortex. Add 100 µl to all wells, including the blanks.

**7 – Incubate at room temperature for 1 hour.**

**8 – Wash immunoplate 5 times (see step 5).**

**9 – Add 100 µl/ well of TMB solution.** Add to all wells, including the blanks.

**10 – Incubate at room temperature (usually 30 - 60 minutes).** You may read the developing blue color at 650 nm and use the data for your calculations.

**11 – Terminate reactions by adding 100 µl 2 N HCI per well.**

**12 – Read absorbance at 450 nm within ten minutes.**

1. **Additional exploratory results**

Table 3. Descriptives of individuals who provided pre NSSI samples vs. those who did not

| **Variable** | **Individuals providing pre NSSI sample (*n* =** **8)**  ***M* (*SD*)** | **Individuals *not* providing pre NSSI sample (*n* =** **43)**  ***M* (*SD*)** |
| --- | --- | --- |
| Age | 30 (8.04) | 22.79 (5.88) |
| Years of education | 11.88 (0.99) | 11.87 (1.52) |
| % psychotropic medication | 62.5 | 62.79 |
| Comorbid diagnoses_a_ | 2.5 (1.6) | 2.19 (1.44) |
| Age at first engagement in NSSI | 15.25 (3.59) | 14.14 (3.59) |
| Years of engagement in NSSI | 14.75 (5.34) | 8.69 (6.27) |
| Number of NSSI acts last month | 8.94 (3.99) | 10.63 (6.81) |
| Pain intensity during NSSI_b_ | 4.12 (2.47) | 4.49 (1.99) |
| Severity of NSSI_c_ (last three months) | 2.25 (0.46) | 2.19 (0.5) |
| Correlation β-endorphin and NSSI urge | r = -0.23 | r = -0.01 |

_a_ assessed with SCID-I interview

_b_ painfulness was rated on a ten-point Likert scale from 1 (no pain) to 11 (worst imaginable pain)

_c_ severity categories: 1=**mild**: superficial cuts, bruise, scratching, 2=**moderate**: not only skin, but also the underlying tissue is damaged, strong bleeding cuts, 2/3 grade burning, 3=**severe**: cuttings until fat tissue, damaged sinews, bone fractures, inner bleeding

**References**

1 Wittchen HU, Wunderlich U, Gruschwitz S. SKID. Strukturiertes Klinisches Interview für DSM-IV Achse I*.* Hogrefe: Göttingen; 1997.

2 Loranger AW, Sartorius N, Andreoli A, Berger P, Buchheim P, Channabasavanna SM, et al. Deutschsprachige Fassung der International Personality Disorder Examination: IPDE*.* WHO: Genf; 1998.

3 Fischer G, Ameis N, Parzer P, Plener PL, Groschwitz R, Vonderlin E, et al. The German version of the self-injurious thoughts and behaviors interview (SITBI-G): a tool to assess non-suicidal self-injury and suicidal behavior disorder. BMC psychiatry. 2014;14(1):265.

4 Spitzer RL, Williams JB, Gibbon M, First MB. Structured Clinical Interview for DSM-III-R Axis II Disorders (SCID-II). Washington, DC: American Psychiatric Association. 1990.

5 Moshagen M, Hilbig BE, Zettler I. Faktorenstruktur, psychometrische Eigenschaften und Messinvarianz der deutschsprachigen Version des 60-Item HEXACO Persönlichkeitsinventars. Diagnostica. 2014.

6 Bohus M, Kleindienst N, Limberger MF, Stieglitz R-D, Domsalla M, Chapman AL, et al. The short version of the Borderline Symptom List (BSL-23): development and initial data on psychometric properties. Psychopathology. 2009;42(1):32-39.

7 Stiglmayr C, Schimke P, Wagner T, Braakmann D, Schweiger U, Sipos V, et al. Development and psychometric characteristics of the Dissociation Tension Scale. Journal of Personality Assessment. 2010;92(3):269-77.

8 Wingenfeld K, Spitzer C, Mensebach C, Grabe HJ, Hill A, Gast U, et al. Die deutsche version des childhood trauma questionnaire (CTQ): Erste befunde zu den psychometrischen Kennwerten. PPmP-Psychotherapie· Psychosomatik· Medizinische Psychologie. 2010;60(11):442-50.

9 Cohen S, Doyle WJ, Skoner DP, Rabin BS, Gwaltney JM. Social ties and susceptibility to the common cold. JAMA. 1997;277(24):1940-44.

10 Wilhelm P, Schoebi D. Assessing mood in daily life. European Journal of Psychological Assessment. 2007;23(4):258-67.

11 Röcke C, Grühn D. German translation of the PANAS-X. Unpublished manuscript, Free University Berlin. 2003.

12 Gratz KL. Risk factors for and functions of deliberate self‐harm: An empirical and conceptual review. Clinical Psychology: Science and Practice. 2003;10(2):192-205.

13 Rallis BA, Deming CA, Glenn JJ, Nock MK. What is the role of dissociation and emptiness in the occurrence of nonsuicidal self-injury? Journal of Cognitive Psychotherapy. 2012;26(4):287-98.

14 APA APA. Diagnostic and statistical manual of mental disorders : DSM-5*.* 5. ed. ed. American Psychiatric Association: Arlington, VA; 2013.

15 Stiglmayr C, Schmahl C, Bremner JD, Bohus M, Ebner-Priemer U. Development and psychometric characteristics of the DSS-4 as a short instrument to assess dissociative experience during neuropsychological experiments. Psychopathology. 2009;42(6):370-74.

16 Engel R, Groves J. Verhaltens-und Erlebensinventar. Deutschsprachige Adaptation des Personality Assessment Inventory (PAI) von LC Morey Göttingen, Hogrefe. 2013.

17 Stein MB, Pinsker-Aspen JH, Hilsenroth MJ. Borderline pathology and the Personality Assessment Inventory (PAI): An evaluation of criterion and concurrent validity. J Pers Assess. 2007;88(1):81-89.
